# Supplementary figures and images for: Vinpocetine alleviates lung inflammation via macrophage inflammatory protein-1β inhibition in an ovalbumin-induced allergic asthma model
Source: PLoS One. 2021 Apr 29;16(4):e0251012. doi: 10.1371/journal.pone.0251012 (PMC8084130; doi:10.1371/journal.pone.0251012)

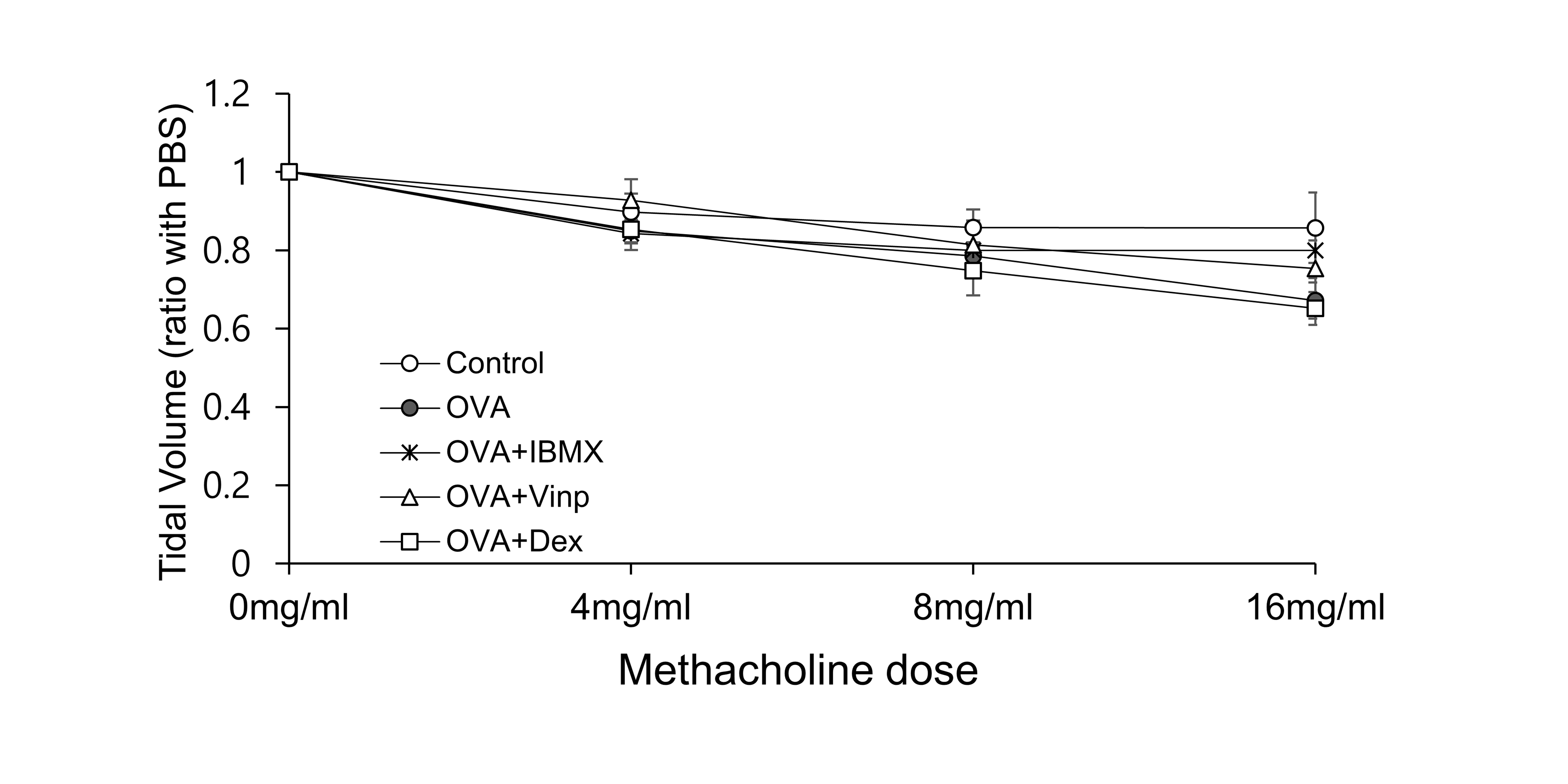

Supplement: S1 Fig — The methacholine test was performed to measure tidal volume. Mice in the OVA, OVA + IBMX, OVA + Vinp, and OVA + Dex groups were exposed to methacholine (4, 8, 16 mg/ml). After methacholine exposure, the tidal volume of the lungs was measured by plethysmography. Data are expressed as mean ± SEM. Statistical analysis was performed using the Student’s t-test, one-way ANOVA, and two-way ANOVA. OVA, ovalbumin; IBMX, 3-isobutyl-1-methylxanthine; Vinp, vinpocetine; Dex, dexamethasone. (TIF) [file pone.0251012.s001.tif]

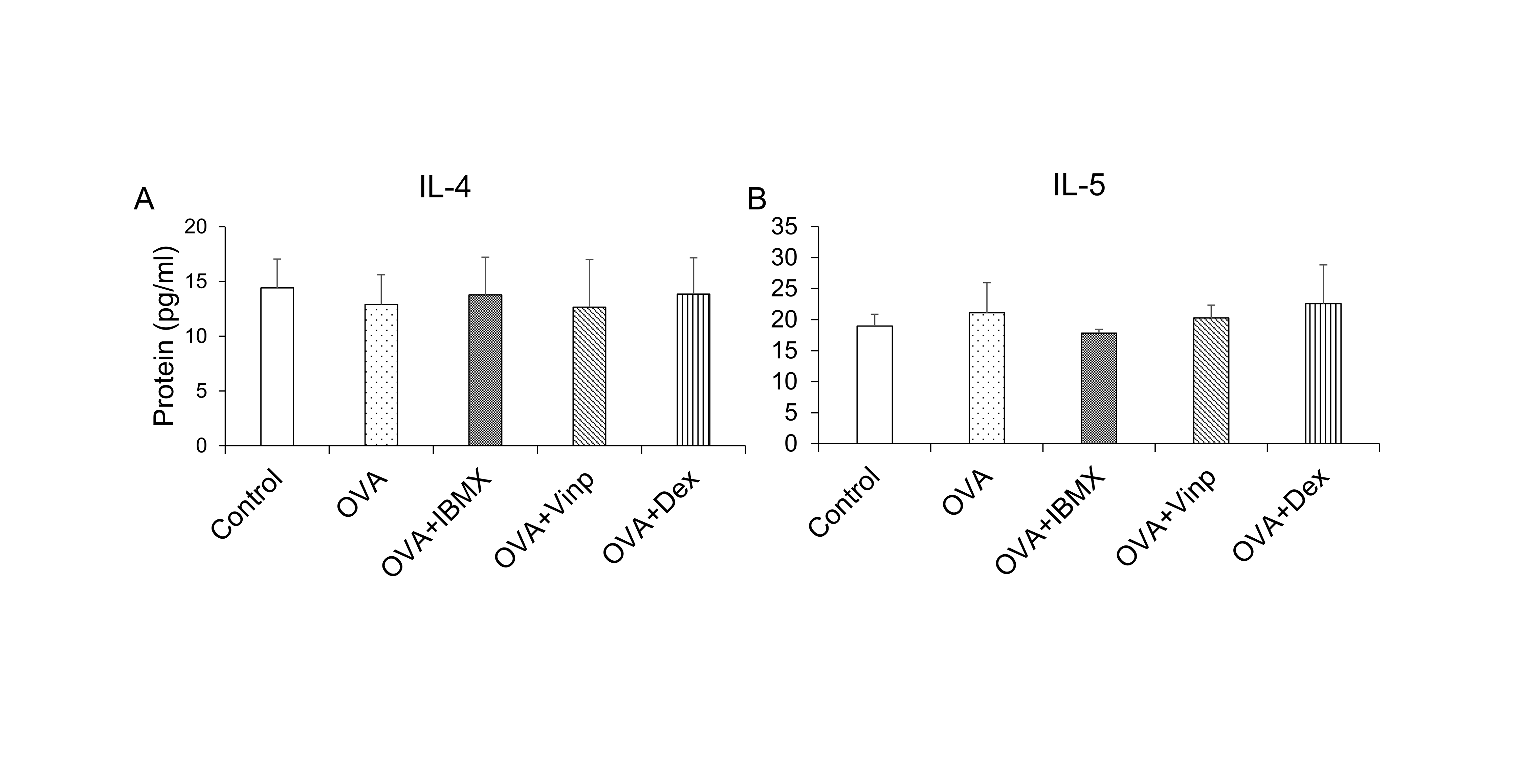

Supplement: S2 Fig — (A) and (B) The release of IL-4 and IL-5 in BALF was measured by ELISA. Data are expressed as mean ± SEM. Statistical analysis was performed using one-way ANOVA. BALF, broncho-alveolar lavage fluid; OVA, ovalbumin; Vinp, vinpocetine; Dex, dexamethasone. (TIF) [file pone.0251012.s002.tif]

PDE1A

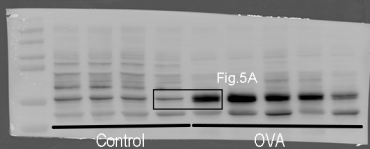

$\beta$ -Actin

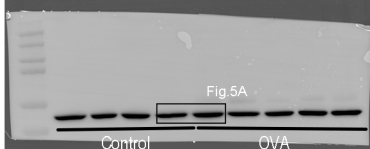

PDE1B

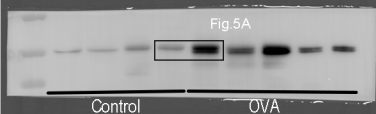

$\beta$ -Actin

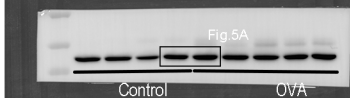

PDE1C

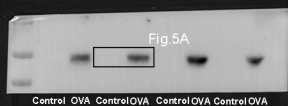

$\beta$ -Actin

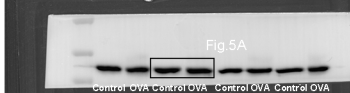

Supplement: S1 Raw images — (PDF) [file pone.0251012.s003.pdf]
