# Supplementary material for: Vinpocetine alleviates lung inflammation via macrophage inflammatory protein-1β inhibition in an ovalbumin-induced allergic asthma model
Source: PLoS One. 2021 Apr 29;16(4):e0251012. doi: 10.1371/journal.pone.0251012 (PMC8084130; doi:10.1371/journal.pone.0251012)
Supplement: Attachment — Submitted filename: Authors response_PLOS ONE.docx [file pone.0251012.s005.docx]

*PLOS ONE*

Manuscript number: PONE-D-20-32932

Title: Vinpocetine alleviates lung inflammation via macrophage inflammatory protein-1β inhibition in an ovalbumin-induced allergic asthma model

Revision date: March 10, 2021

We would like to thank you and the reviewers for the comments on our manuscript, which is entitled as “Vinpocetine alleviates lung inflammation via macrophage inflammatory protein-1β inhibition in an ovalbumin-induced allergic asthma model” We revised the manuscript according to the reviewer’s helpful comments. We made red color revised part. We hope that this revised manuscript can be suitable for PLOS ONE’s publication criteria.

Review Comments to the Author

Reviewer #1: NA

Reviewer #2: The present manuscript adressed all my comments. Especially the material and method part is clearly improved. However, the discussion part still strongly focuses on the own results, here a comparison to other PDE inhibitors would be desirable.

Author’s response

Thank you for your helpful comment. We revised the discussion part to discuss not only PDE 1 based on our data but also another PDE subtype using other previous studies using different PDE inhibitors in asthma models

[Line 332-336]

It was known that PDE inhibitors have effect on respiratory disease such as asthma and chronic obstruction pulmonary disease [22, 39]. In particular, PDE 3, 4, and 7 inhibitors are known to be effective in mouse models of asthma from previous studies [22]. However, studies on asthma using the PDE 1 inhibitor in allergic asthma mice model have not been studied yet.

[Line 346-351]

Previous research reported that PDE4 inhibition was shown to have an effect on the attenuation of airway resistance in asthma mice model [43]. PDE3 inhibitor and PDE1/4 dual inhibitor, has been reported to cause the inhibition of the ovalbumin‐induced bronchoconstriction [44]. These previous studies supported that PDE inhibition can alleviated the increased airway resistance. Through this study, we showed that PDE 1 inhibitor can ameliorated the airway resistance increased in asthma mice model.

[Line 357-364]

It was reported that PDE 4 inhibitor reduced the number of eosinophils, neutrophils, and lymphocytes in BALF [47]. …………….………..The effect on inflammatory cells was similar to previous reports using PDE 4 inhibitors.

[Line 378-380]

In previous research, among the PDE inhibitors, PDE 4 inhibitors were reported to affect the inhibition of inflammatory cytokines of BALF (IL-4, 5 and 13) [43].

[Line 417-421]

Through previous studies, it was already demonstrated that PDE4A, 4C, and 4D expression level was increased in allergic pulmonary inflammatory conditions [57, 58]. These research showed the PDE4 was associated with asthmatic conditions. In this study, PDE1A, 1B, and 1C mRNA expression levels and protein expression levels in the lungs were all increased. These results show that PDE1 expression is also associated with asthma.

[References]

22. Page CP. Phosphodiesterase inhibitors for the treatment of asthma and chronic obstructive pulmonary disease. Int Arch Allergy Immunol. 2014;165(3):152-64.http://doi.org/10.1159/000368800 PMID: 25532037

39. Kwak HJ, Nam JY, Song JS, No Z, Yang SD, Cheon HG. Discovery of a novel orally active PDE-4 inhibitor effective in an ovalbumin-induced asthma murine model. Eur J Pharmacol. 2012;685(1):141-8.https://doi.org/10.1016/j.ejphar.2012.04.016 PMID: 22554769

43. Kim SW, Kim JH, Park CK, Kim TJ, Lee SY, Kim YK, et al. Effect of roflumilast on airway remodelling in a murine model of chronic asthma. Clin Exp Allergy. 2016;46(5):754-63.https://doi.org/10.1111/cea.12670 PMID: 26542330

44. Myou, Fujimura, Kurashima, Tachibana, Hirose, Nakao. Effect of aerosolized administration of KF19514, a phosphodiesterase 4 inhibitor, on bronchial hyperresponsiveness and airway inflammation induced by antigen inhalation in guinea-pigs. Clin Exp Allergy. 2000;30(5):713-8.https://doi.org/10.1046/j.1365-2222.2000.00782.x PMID: 10792364

47. Sun J-g, Deng Y-m, Wu X, Tang H-f, Deng J-f, Chen J-q, et al. Inhibition of phosphodiesterase activity, airway inflammation and hyperresponsiveness by PDE4 inhibitor and glucocorticoid in a murine model of allergic asthma. Life Sci. 2006;79(22):2077-85.https://doi.org/10.1016/j.lfs.2006.07.001 PMID: 16875702

57. Tang H-F, Song Y-H, Chen J-C, Chen J-Q, Wang P. Upregulation of phosphodiesterase-4 in the lung of allergic rats. Am J Respir Crit Care Med. 2005;171(8):823-8.http://doi.org/10.1164/rccm.200406-771OC PMID: 15665325

58. Trian T, Burgess JK, Niimi K, Moir LM, Ge Q, Berger P, et al. β2-agonist induced cAMP is decreased in asthmatic airway smooth muscle due to increased PDE4D. PLoS One. 2011;6(5):e20000.http://doi.org/10.1371/journal.pone.0020000 PMID: 21611147
